# SCF-KIT signaling induces endothelin-3 synthesis and secretion: Thereby activates and regulates endothelin-B-receptor for generating temporally- and spatially-precise nitric oxide to modulate SCF- and or KIT-expressing cell functions

**DOI:** 10.1371/journal.pone.0184154

**Published:** 2017-09-07

**Authors:** Lei L. Chen, Jing Zhu, Jonathan Schumacher, Chongjuan Wei, Latha Ramdas, Victor G. Prieto, Arnie Jimenez, Marco A. Velasco, Sheryl R. Tripp, Robert H. I. Andtbacka, Launce Gouw, George M. Rodgers, Liansheng Zhang, Benjamin K. Chan, Pamela B. Cassidy, Robert S. Benjamin, Sancy A. Leachman, Marsha L. Frazier

**Affiliations:** 1 Department of Sarcoma, University of Texas M D Anderson Cancer Center, Houston, Texas, United States of America; 2 Department of Epidemiology, University of Texas M D Anderson Cancer Center, Houston, Texas, United States of America; 3 ARUP Laboratories, Salt Lake City, Utah, United States of America; 4 Research Information Services & Technology, University of Texas M D Anderson Cancer Center, Houston, Texas, United States of America; 5 Pathology, University of Texas M D Anderson Cancer Center, Houston, Texas, United States of America; 6 Vel-Lab Research, Missouri City, Texas, United States of America; 7 Department of Surgery, University of Utah, Salt Lake City, Utah, United States of America; 8 Department of Internal Medicine, University of Utah, Salt Lake City, Utah, United States of America; 9 Department of Hematology & Oncology, The Second Hospital of Lanzhou University, Lanzhou, Gansu, P. R. China; 10 Department of Biology, University of Utah, Salt Lake City, Utah, United States of America; 11 Department of Dermatology, University of Utah, Salt Lake City, Utah, United States of America; 12 Graduate School of Biomedical Sciences, University of Texas M D Anderson Cancer Center, Houston, Texas, United States of America; Max Delbruck Centrum fur Molekulare Medizin Berlin Buch, GERMANY

## Abstract

We demonstrate that SCF-KIT signaling induces synthesis and secretion of endothelin-3 (ET3) in human umbilical vein endothelial cells and melanoma cells *in vitro*, gastrointestinal stromal tumors, human sun-exposed skin, and myenteric plexus of human colon post-fasting *in vivo*. This is the first report of a physiological mechanism of ET3 induction. Integrating our finding with supporting data from literature leads us to discover a previously unreported pathway of nitric oxide (NO) generation derived from physiological endothelial NO synthase (eNOS) or neuronal NOS (nNOS) activation (referred to as the KIT-ET3-NO pathway). It involves: (1) SCF-expressing cells communicate with neighboring KIT-expressing cells directly or indirectly (cleaved soluble SCF). (2) SCF-KIT signaling induces timely local ET3 synthesis and secretion. (3) ET3 binds to ETBR on both sides of intercellular space. (4) ET3-binding-initiated-ETBR activation increases cytosolic Ca^2+^, activates cell-specific eNOS or nNOS. (5) Temporally- and spatially-precise NO generation. NO diffuses into neighboring cells, thus acts in both SCF- and KIT-expressing cells. (6) NO modulates diverse cell-specific functions by NO/cGMP pathway, controlling transcriptional factors, or other mechanisms. We demonstrate the critical physiological role of the KIT-ET3-NO pathway in fulfilling high demand (exceeding basal level) of endothelium-dependent NO generation for coping with atherosclerosis, pregnancy, and aging. The KIT-ET3-NO pathway most likely also play critical roles in other cell functions that involve dual requirement of SCF-KIT signaling and NO. New strategies (e.g. enhancing the KIT-ET3-NO pathway) to harness the benefit of endogenous eNOS and nNOS activation and precise NO generation for correcting pathophysiology and restoring functions warrant investigation.

## Introduction

Nitric oxide (NO) is produced by three isoforms of NO synthase (NOS), neuronal NOS (nNOS, type 1), endothelial NOS (eNOS, type 3), and inducible NOS (iNOS, type 2). eNOS and nNOS are expressed mainly in endothelial cells and neurons respectively and their activation is Ca^2+^-dependent. The constitutive low level of NO generated by eNOS and or nNOS mediates diverse cell-specific physiological functions [1,2]. Unlike eNOS and nNOS, activation of iNOS does not depend on Ca^2+^, and is mainly induced by inflammation. iNOS activation typically generates a burst of excessive NO production, triggers apoptosis, induces cell death, and has been associated with degenerative diseases [3].

The diverse physiological role of NO depends on target cell types, downstream signaling effector molecules, micro environmental cues (presence or absence of growth factors or cytokines), the source of NO generation (by eNOS, or nNOS, or iNOS), constitutive *vs*. burst NO production, and concentration (nanomolar *vs*. micromolar NO levels). The above variables contribute to the different effects of NO in proliferation *vs*. differentiation [4], survival *vs*. apoptosis [5], and neuroprotection *vs*. neurodegeneration [6]. Therefore, timely delivery of precise amount of NO generated by the specific NOS activation is critical for cell-specific functions, maintaining homeostasis, and survival.

Our goal is to study the upstream signaling and regulation of eNOS and nNOS activation with the hope to harness the physiological protective benefit of NO, correct underlying pathophysiology, and restore functions. We studied endothelium because endothelium impairment is associated with many serious human diseases.

Vascular tone is collectively regulated by the nervous, renin-angiotensin, endocrine, renal, and endothelin system [7–10], which consists of three structurally similar peptide ligands, endothelin-1 (ET1), endothelin-2 (ET2), and endothelin-3 (ET3), and two receptors, endothelin-A-receptor (ETAR) and endothelin-B-receptor (ETBR), which is selectively expressed and mediates pleiotropic cell-specific diverse functions [9,10]. Endothelium-dependent NO generation can be increased by three main stimuli: shear force (e.g. increased blood flow), acetylcholine, and ETBR activation. ETBR belongs to the family of G protein-coupled receptors. Ligand binding to ETBR leads to an increase of cytosolic Ca^2+^ [11,12], followed by Ca^2+^-dependent cell-specific eNOS and nNOS activation and temporally- and spatially-precise NO generation [10–12]. The upstream regulation of ETBR activation is not well understood and remain one of the focus of this study.

NO bioavailability represents the difference between total endothelium-dependent-NO generation and NO breakdown (e.g. oxidative stress and or inhibition by physiological NOS inhibitors), and the major and immediate physiological compensatory mechanisms for insufficient NO bioavailability is to enhance eNOS activation and increase total endothelium-dependent-NO generation [13–15]. When the high demand (exceeding basal level) of endothelium-dependent NO generation is not fulfilled, NO bioavailability becomes insufficient, vascular homeostasis crumbles, and diseases ensue. Atherosclerosis, normal pregnancy, and physiological aging share a common denominator of high demand for endothelium-dependent NO generation. The compensatory mechanisms of these three conditions provides insights, so a brief background is described below.

Atherosclerosis is the major cause for developing vascular events [2,13–18] including ischemic heart disease, heart failure, stroke, and peripheral arterial occlusive disease (PAOD). A genetic manipulation study comparing double (apolipoprotein E and eNOS) knockout with single (apolipoprotein E) knockout model demonstrate that eNOS deficiency increases atherosclerosis in Western-type diet-fed single (apolipoprotein E) knockout mice and introduces an array of cardiovascular complications, including myocardial infarction, heart failure, and spontaneous aortic aneurysm and dissection [18]. Endothelium-dependent NO generation derives from eNOS activation and mediates many critical functions including: (**i**) Vasodilation to counteract untoward vasoconstriction [19]. (**ii**) Anti-apoptosis. (**iii**) Inhibition of platelet and leukocyte aggregation. (**iv**) Attenuation of ET1-induced signaling [20,21] and negative regulation of ET1 synthesis [22]. Elevated plasma ET1 level is pro-inflammatory, pro-fibrotic, and is detrimental. (**v**) A critical unique role in maintaining the vascular smooth muscle cells (VSMCs) in a non-proliferative state thus prevent acceleration of pre-existing or developing new atherosclerosis. [2,13,16]. Above studies [2,13–18] indicate clearly that endothelium-dependent NO generation plays a critical protective anti-atherogenic role.

Both endothelial-specific ETBR-knockout (ETBR^–/–^) [23] and rescued-ETBR^–/–^mice [24] manifest significantly decreased endothelium-dependent NO generation and elevated plasma ET1 level despite compensated normal blood pressure (BP), thus provide unequivocal evidences indicating a critically important physiological role of ETBR in the activation of eNOS and NO generation in endothelium.

Normal pregnancy is associated with increased maternal blood volume and cardiac output, decreased systemic vascular resistance, and increased release of vasodilators. During pregnancy, eNOS is upregulated in uterine and systemic arteries, and circulating NO level is increased [25]. Enhancement of ETBR-mediated microvascular relaxation contributes to the decreased vasoconstriction and vascular resistance during pregnancy [26]; conversely, down-regulation of microvascular endothelial ETBR is a central vascular mechanism leading to preeclampsia [27]. These studies indicate that ETBR-mediated physiological NO generation in endothelium not only exists as demonstrated by the ETBR^–/–^mouse model [23,24], but also plays a critical role in increasing endothelium-dependent NO generation when demand exceeds basal level, such as during pregnancy, for maintaining homeostasis.

Normal aging is characterized by a progressive reduction of NO bioavailability [28–32]. In kidney, medullary vasodilation depends on NO and, to a lesser extent, on prostaglandin. In senescent rats, medullary ET3 content increases by 3.4-fold comparing to healthy young adult control rats (*P* < 0.05) and manifests preservation of local NO levels in medulla, whereas cortex ET3 remains unchanged and manifests decreased local NO level during normal aging in rats [33]. These results indicate that the 3.4-fold increase of ET3 leading to enhanced activation of ETBR and increased endothelium-dependent NO generation is a physiological compensatory mechanism against age-related reduction of NO bioavailability.

ET3 knockout mice die with an average of 21 days after birth indicating its vital physiological roles postpartum. ET3 is the only ligand that can differentiate ETBR from ETAR [9]. ET3 is dedicated to binding to ETBR nearly exclusively at physiological concentrations with 100 times greater binding affinity toward ETBR than toward ETAR [9,34], and functions locally [35,36]. ET3 binds to ETBR with equal affinity as ET1 [37], thus can induce comparable vasodilation as ET1. To be more precise, quantitative analysis with direct comparison indicate that the maximal vasodilation effect by ET1 is about 70% of that by ET3 [38]. ET1 and ET2 can bind to both ETAR and ETBR. So, in sharp contrast to ET1, ET3 induces vasodilation with negligible vasoconstriction at physiological concentrations.

The capacity of ET3-ETBR signaling in eNOS induction and NO generation is well documented *in vivo* [39,40] and *in vitro* [12,38,41–43]. Likewise, ample reports have demonstrated nNOS induction and NO generation by ET3-ETBR signaling [44–52]. Ligand availability plays a critical rate-limiting regulatory role in membrane receptor activation. But the upstream mechanism of genesis and regulation of ET3 remain unknown.

We are intrigued by the overlapping function and dual requirement of both stem cell factor (SCF)-KIT signaling and NO in multiple functions (refer to the last Section in Results & Discussion for examples and details). So, we explored KIT-mediated downstream signaling as the first step toward our goal. KIT is a type III receptor tyrosine kinase. SCF exists in a membrane-bound form and a soluble form for longer-range signal transmission. KIT is expressed on stem/progenitor cells including bone marrow multipotent stem cells, endothelial progenitor cells (EPCs), resident cardiac stem/progenitor cells [53,54], resident neuronal stem/progenitor cells [55], resident melanocyte progenitor cells [56,57], and mature cells including endothelium, interstitial cells of Cajal (ICCs), melanocytes, glial cells (e.g. astrocytes), pancreatic islet β-cells, germ cells, monocytes, natural killer cells, and mast cells.

We demonstrate that ET3 is a downstream target of SCF-KIT signaling and discover a previously unreported cell-communication-initiated tightly-controlled physiological mechanism of cell-specific eNOS and or nNOS activation leading to temporally- and spatially-precise NO generation in either KIT-expressing and or neighboring SCF-expressing cells (hereafter referred to as the KIT-ET3-NO pathway). We demonstrate that the KIT-ET3-NO pathway plays a critical role in fulfilling the high demand of endothelium-dependent NO generation for compensating pathophysiology (e.g. atherosclerosis) or normal physiology (e.g. pregnancy or aging).

## Materials and methods

### Cells, tissues, and tumor specimens

Gastrointestinal stromal tumors (GISTs) and normal human colon tissue specimens were obtained with consent according to MD Anderson Institutional Review Board-approved laboratory protocol LAB02-433. Normal human adult testis specimens were purchased from Asterand (Detroit, MI, USA). Unused surgical specimens containing normal human skin and skin punch biopsy specimens were obtained with consent according to University of Utah Institutional Review Board-approved protocol 10924 and 7916 respectively. Human umbilical vein endothelial cells (HUVECs) were purchased from Cambrex Bio Science (Walkersville, MD, USA) and cultured as recommended by the vendor. WM793 melanoma cell line is a subclone of American Type Culture Collection WM793 and was provided by Dr. Suhendan Ekmekcioglu at MD Anderson Cancer Center, University of Texas. GIST882 cell line was provided by Dr. Jonathan Fletcher at Brigham and Women's Hospital, Harvard Medical School.

### KIT sequence analysis

The primer sequences and genomic and cDNA sequencing analysis of *KIT* were described previously [58].

### Microarray analysis

Precipitated total RNA of GISTs was suspended in diethylpyrocarbonate treated water. Contaminated DNA was removed by using a DNA-Free kit (Ambion, Austin, TX, USA). RNA samples were analyzed for RNA integrity using an Agilent 2100 Bioanalyzer (Agilent Technologies, Palo Alto, CA, USA). cDNA was prepared as described previously [59]. Hybridization to microarrays was performed using a human oligonucleotide spotted glass array with 18,861 60-mer oligos and controls produced in the Wiegand Radiation Oncology Microarray Core Facility at MD Anderson Cancer Center. Hybridization was carried out for 16 hours at 50°C. Slides were washed as described previously [59] and scanned with an ArrayWorx autoscanner (Applied Precision Inc., Issaquah, WA, USA). Quantified image data were processed using the statistical software package Splus 6 (Insightful, Seattle, WA, USA). Local estimated background signal intensity was subtracted from raw total signal intensity for each feature (spot). A logarithm-2–transformation was applied to the background-corrected signals. Within each channel, cy3 and cy5, on each array, the logarithm-2–transformed signals were normalized to the 75th percentile of the signal intensity. Signals were filtered according to the requirement that the signal-to-noise ratio be greater than 2 in at least 80% of the arrays in each group. The ratio of the logarithm-2–based data for the two samples was used for further analysis to identify differentially regulated genes.

### Enzyme-linked immunosorbent assay (ELISA)

SCF was obtained from Calbiochem (San Diego, CA). Enzyme-linked immunosorbent assay (ELISA) was performed using an ET3 assay kit according to the manufacturer’s recommendations (IBL-America, Minneapolis, MN, USA). Optical density was determined at 450 nm using a Gemini EM ELISA reader (SpectraMax, Molecular Devices, Downingtown, PA, USA).

### Immunohistochemistry (IHC)

Antibodies include: ETBR (Abacam, Cambridge, MA), human endothelin-converting enzyme-1 (ECE-1) (Invitrogen, Carlsbad, CA), ET3 (Abnova, Taipei, Taiwan), BigET3 (BACHEM, Torrance, CA), polyclonal antibodies against tyrosine-phosphorylated KIT-peptides (Biosource International, Camarillo, CA), Melan-A (Dako, Carpenteria, CA), pan-KIT antibody (recognizing the non-phosphorylated last 14 amino acids of the *C*-terminal end of KIT) (Dako, Carpenteria, CA), and secondary antibodies (rabbit anti-mouse and mouse anti-rabbit Fab fragments) (Dako, Carpenteria, CA). Primary antibodies were diluted 1:100 and incubated at 4°C overnight for frozen-section IHC. For *in situ* IHC, WM793 cells were grown in 4-well Chamber Tissue Culture Treated Glass Slides (Fisher Scientific Inc., Pittsburgh, PA) as monolayers. Cells were fixed with glutaraldehyde. For Paraffin-embedded-section, antigen retrieval is not necessary for KIT, the primary antibody was diluted 1:100, and incubated at 37°C for 32 minutes. Melan-A was retrieved using citrate buffer (pH 6.0), the primary antibody was diluted 1:200, and incubated at 37°C for 40 minutes. ET3, ETBR, and BigET3 were retrieved using target retrieval solution pH 6 (Dako, Carpinteria, CA) for 20 minutes at sub boiling temperature, followed by primary antibodies against ET3, ETBR, BigET3 at dilution of 1:200, 1:100, and 1:1000 respectively, at 4°C overnight. ECE-1 was retrieved using antigen retrieval solution pH 6 (Vector Labs, Burlingame, CA), followed by primary antibody at 1:100 dilution at 4°C overnight. Antibodies were visualized with VECTASTAIN^®^ Elite ABC kits (Vector Labs, Burlingame, CA), and cells were counterstained with hematoxylin.

## Results and discussion

### Microarray analysis comparing an extremely aggressive verse an indolent gastrointestinal stromal tumor (GIST): An 8.5-fold up-regulation of endothelin-3 (ET3) expression in the aggressive GIST

GISTs arise from ICCs. Dominant activating-mutations of either *KIT* or *PDGFR-A* can lead to GIST. KIT signaling is critical and sufficient for GIST development [60]. An activating-mutation of *KIT* accounts for approximately 75–79%, and activating-mutation of *PDGFR-A* accounts for approximately 7–21% of all adult GISTs [61–63]. Imatinib mesylate (Gleevec^®^, Glivec^®^) [64], a selective inhibitor of chimeric BCR-ABL (and ABL), KIT, PDGFR-α, and PDGFR-β, is the FDA recommended first line treatment for GIST. Evolution from heterozygous to homozygous *KIT* mutation in GISTs correlates with increased mitotic count, a striking fourfold increase in topoisomerase II proliferative index, and significant tumor progression [63], so, we paired such a highly aggressive GIST harboring a homozygous Trp557Gly *KIT* mutation (showing 24 mitotic figures per 50 high-power fields and a topoisomerase II proliferative index of 30.8%) with an indolent GIST harboring a 6-bp internal tandem duplication in *KIT* exon 9 (A502_Y503 dup) (showing 3 mitotic figures per 50 high-power fields and a topoisomerase II proliferative index of 8%). Both GISTs had an initial excellent response to imatinib and later developed resistance due to the same second new single nucleotide missense *KIT* mutation (1982T→C, Val654Ala) resulting in abrogation of imatinib binding to KIT [58,65]. Both GISTs show normal cytogenetic profiles (data not shown). The initial excellent response to imatinib and the later progression due to imatinib-resistance confirm that the constitutively activated KIT signaling is the driving force of both GISTs, and direct comparison of highly aggressive versus indolent GISTs should provide insight to SCF-KIT signaling and help to identify downstream target genes.

Genes that are differ by two-fold or more are listed in Table 1. A 2.1-fold of down-regulation of *PDGFR-A* is observed in the highly aggressive GIST (Table 1). KIT and PDGFR-α belong to type III receptor tyrosine kinase family and share structural similarity. KIT and PDGFR-α share functional redundancy as evidenced by three observations: (i) human *KIT* and *PDGFR-A* are genetically closely linked on chromosome *4q12*, (ii) activating mutation of either *KIT* or *PDGFR-A* can lead to the same malignancy of GISTs, and (iii) our microarray finding of down-regulation of *PDGFR-A* in a highly aggressive GIST exhibiting constitutive activation of *KIT* further supports functional redundancy between KIT and PDGFR-α.

**Table 1:** Microarray analysis comparing a highly aggressive GIST with an indolent GIST: A selective list of target genes whose expression was up- or down-regulated by KIT signaling.

ACC	Symbol	Description	Fold change
NM_000114	EDN3 (ET3)	endothelin-3	8.48
NM_001565	CXCL10	chemokine (C-X-C motif) ligand 10	4.95
NM_001452	FOXF2	forkhead box F2	3.09
NM_005596	NFIB	nuclear factor I/B	2.99
NM_175866	KIS	kinase interacting with leukemia-associated gene (stathmin)	2.44
NM_018664	SNFT	Jun dimerization protein p21SNFT	2.31
NM_000362	TIMP3	tissue inhibitor of metalloproteinase 3	2.22
NM_002198	IRF1	interferon regulatory factor 1	2.05
NM_006206	PDGFR-A	platelet-derived growth factor receptor, alpha polypeptide	-2.11
NM_006769	LMO4	LIM domain only 4	-2.21
NM_004440	EPHA7	EphA7	-2.70
NM_001946	DUSP6	dual specificity phosphatase 6	-2.92
NM_004490	GRB14	growth factor receptor-bound protein 14	-6.86

A striking 8.5-fold up-regulation of ET3 expression in the aggressive GIST stands out as the major differences between these two GISTs and suggests that ET3 is a downstream target of SCF-KIT signaling (Table 1). This result was confirmed using a different preparation of oligonucleotide spotted glass arrays, and was reproducible in other pairs of GISTs (data not shown). We proceed to validate this microarray finding.

### The SCF-KIT signaling induces endothelin-3 (ET3) synthesis and secretion in human umbilical vein endothelial cells (HUVECs) and human melanoma cells *in vitro*

**Fig 1:**
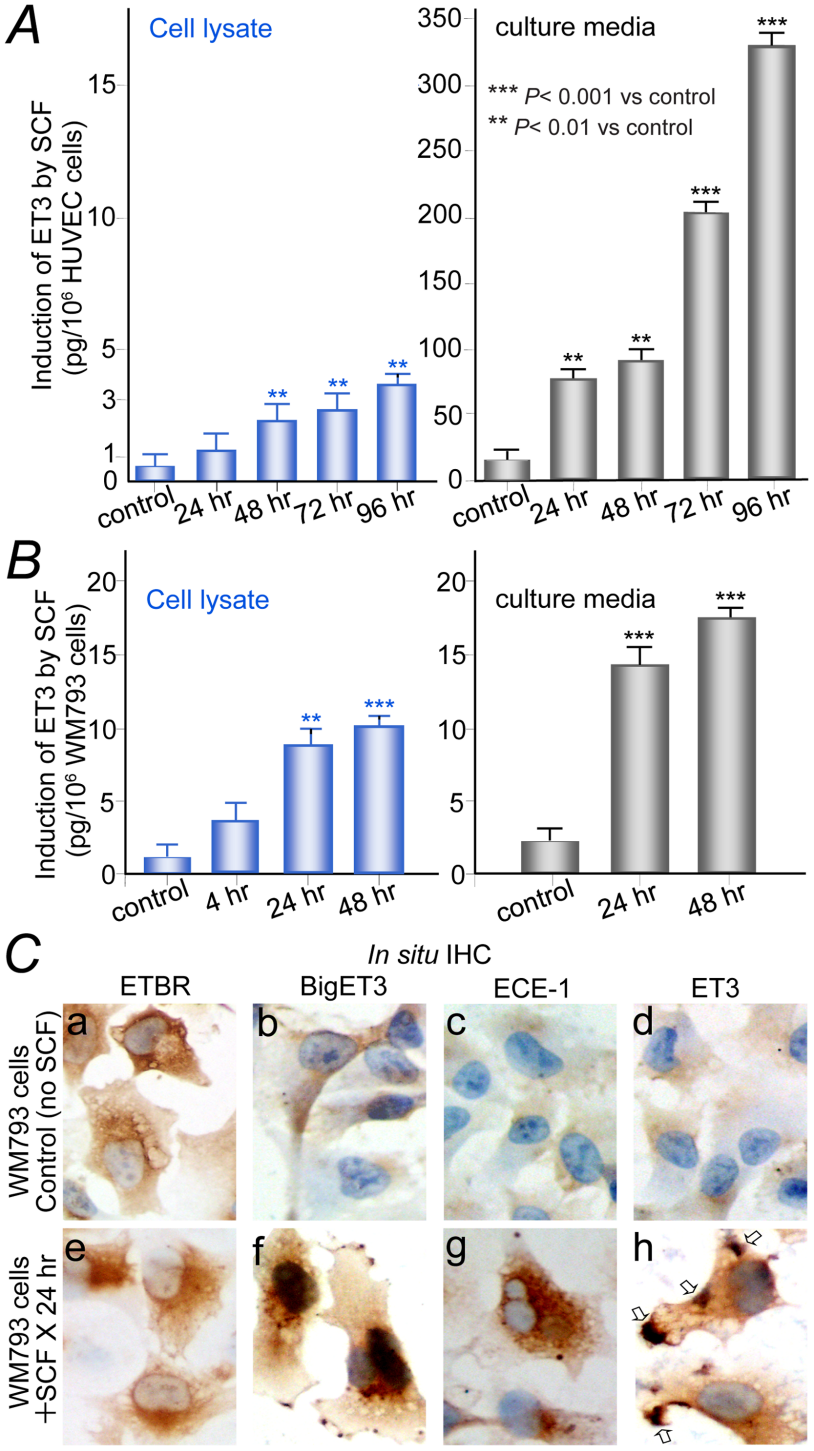
Enzyme-linked immunosorbent assay (ELISA) and *in situ* immunohistochemistry demonstrate induction of endothelin-3 by SCF-KIT signaling. (***A***), HUVECs responded to SCF with significant synthesis of ET3 (left panel) and secretion of ET3 in media (right panel). (***B***), WM793 melanoma cells responded to SCF with significant synthesis (left panel) and secretion of ET3 in serum-free culture medium (right panel). (***C***), *In situ* IHC. Top panels, control WM793 cells without SCF stimulation, more than 95% cells exhibited completely negative staining for BigET3, ECE-1, or ET3. Lower panels, WM793 cells after stimulation with SCF (100 ng/ml) for 24 hours. ETBR expression remained unchanged (*a* and *e*). Robust induction of BigET3 (*f*), ECE-1 (*g*), ET3 (*h*), and clusters of sub-membranous ET3 before secretion (*h*, open arrows) are observed in more than 95% of cells comparing to the respective controls (*b*, *c*, *d*).

In response to SCF, HUVECs showed significant (*P* < 0.001) secretion of ET3 (325 pg/million cells) in media after *in vitro* culture for 96 hours (Fig 1A, right panel). WM793 melanoma cells express KIT, and sequencing analysis shows wild-type *KIT* without mutation (data not shown). WM793 melanoma cells also responded to SCF with accumulation of significant ET3 (17 pg/million cells in serum-free culture medium and 10 pg/million cells in cell lysate) after *in vitro* culture for 48 hours (Fig 1B). GIST882 cells (harboring constitutively activated *KIT* due to K642E mutation in exon 13) demonstrated constitutive synthesis and secretion of ET3 with or without SCF stimulation (data not shown).

*In situ* IHC showed that ETBR expression in WM793 was abundant and unchanged with or without SCF stimulation (Fig 1Ca and 1Ce). Without SCF in culture media, more than 95% of cells exhibited completely negative staining for BigET3 (ET3 precursor), ECE-1, and ET3 respectively (Fig 1Cb–1Cd). After addition of SCF to the culture media for 24 hours, we observed strong cytoplasmic staining of BigET3 (Fig 1Cf), ECE-1 (Fig 1Cg), and ET3 (Fig 1Ch) in > 95% of cells. ET3 exhibited clusters of sub-membranous staining before secretion (Fig 1Ch, arrows).

ET3 induction is not part of the known documented KIT signaling pathways (i.e. Ras/Erk, PI3-Kinase/Akt, Src, PLCγ, and JAKs/STAT) [66–68]. Being a 21-amino acids secretory peptide ligand, ET3 is capable of autocrine (or intracrine) and paracrine binding to ETBR on both KIT-expressing and SCF-expressing cells that outline the intercellular space. Membrane receptor kinases activation is mainly controlled and regulated by ligand availability.

In conclusion, we demonstrate induction of ET3 by SCF-KIT signaling using microarray analysis *in* GISTs in *vivo*, using *in situ* IHC direct visualization, and using ELISA in KIT-expressing HUVECs and WM793 melanoma cells *in vitro*. This is the first report of a physiological mechanism of ET3 induction in humans. Our finding of induction of ET3 is important because of its capacity to control and regulate the activation of ETBR (refer to Introduction for background of ETBR and endothelin system).

### The KIT-ET3-NO pathway: A new mechanism of endogenous endothelial nitric oxide synthase (eNOS) and neuronal nitric oxide synthase (nNOS) activation leading to temporally- and spatially-precise nitric oxide (NO) generation

SCF-expressing-cells (e.g. keratinocytes, enteric neurons, VSMCs, Sertoli cells of testis, and most neurons in brain) [69–71] are in close proximity to KIT-expressing cells (e.g. melanocytes, ICCs, endothelium, germ cells, glial cells respectively) [53–57], and this pattern suggests a ligand-receptor relationship and a unique capacity to communicate directly and continuously. Nearly all KIT-expressing cells and their neighboring SCF-expressing-cells express ETBR albeit at wildly different levels [9,35,72]. SCF can also be cleaved from stromal cells by matrix metalloproteinase-9 to serves as the ligand for activation of KIT-expressing bone marrow stem cells or resident progenitor cells for exiting from the quiescent niche [73].

Our finding of induction of ET3 by SCF-KIT signaling represents a missing link, and a belated finding such that many studies of ETBR and NO have already been reported. Our finding enables us to connect seemingly unrelated, isolated, and standing-alone reports and formulate a novel concept. Although we have cited many references, most are for background and completeness. Excluding background references, rather than reinventing the wheels, here we cite the following three reports as the supporting essential data from literature: (**i**) Emori *et al*. 1991 [41], (**ii**) Bagnall *et al*. 2006 [23], (**iii**) Mathison & Israel 2002 [46]. Connecting these supporting essential data with our results leads to the discovery of a new pathway of endogenous eNOS and nNOS activation and NO generation.

In conclusion, integration of background information, above rationales, supporting essential data from the above three reports in literature, and our results together identifies a previously unreported pathway of cell communication-initiated and SCF-KIT signaling-orchestrated cell-specific activation of eNOS and or nNOS (in KIT-expressing and or neighboring SCF-expressing cells) leading to temporally- and spatially precise NO generation for modulating KIT- and or SCF-expressing cell functions for maintaining homeostasis (hereafter referred as the KIT-ET3-NO pathway). It involves the following six essential consecutive steps. (**i**) Activation of SCF-KIT signaling. The SCF-expressing cells can communicate with neighboring KIT-expressing cells directly or indirectly (by soluble SCF cleaved by metalloproteinase) to activate the SCF-KIT signaling. (**ii**) Induction of ET3. Activation of SCF-KIT signaling induces ET3 synthesis and secretion. Being tightly regulated by cell communication-initiated SCF-KIT signaling, ET3 synthesis and secretion is temporally- and spatially-precise and local regional. (**iii**) A unique and singly dedicated role of ET3. Secretory ligand ET3 can bind to ETBR on both sides of intercellular space, meaning both KIT- and neighboring SCF-expressing cells. ET3 binds nearly exclusively to ETBR with very low binding affinity towards ETAR. (**iv**) ETBR activation. Ligand binding of ET3 to ETBR results in cytosolic Ca^2+^ increase, leads to Ca^2+^-dependent cell-specific eNOS and or nNOS activation. (**v**) Temporally- and spatially-precise NO generation derived from cell-specific eNOS and or nNOS activation. The NO thus generated can act as an intracellular signaling molecule and can also diffuse into neighboring cells within 100 μM in a distance- and concentration-dependent fashion. Some KIT-expressing cells (e.g. ICCs, and melanocytes) and some SCF-expressing cells (e.g. VSMCs) do not express NOS, and rely on the NO diffused from their neighboring cells. (**vi**) Physiological role. NO can initiate the NO/cGMP pathway, controlling transcriptional factors, and or other mechanisms to modulate diverse SCF-expressing and or KIT-expressing cell-specific functions for maintaining homeostasis.

#### Endothelium is endowed with the capacity to activate the KIT-ET3-NO pathway when the demand for endothelium-dependent NO generation exceeds basal level for vasodilation and maintaining vascular homeostasis

VSMCs express SCF and predominately ETAR with a very low level of expression of ETBR [9,72,74,75], whereas endothelium expresses KIT and ETBR exclusively without ETAR [9,12,74]. In blood vessels, SCF-expressing VSMCs can communicate with neighboring KIT-expressing endothelium, activate SCF-KIT signaling in endothelium, and fulfill the first of the six essential sequential steps of the KIT-ET3-NO pathway. Our results demonstrated induction of ET3 synthesis and secretion *in vivo* in GISTs (Table 1) using microarray analysis and *in vitro* in HUVECs and WM793 melanoma cells (Fig 1), thus fulfill the second essential sequential step of the KIT-ET3-NO pathway. Since VSMCs express very low level of ETBR, so, the SCF-KIT signaling-induced ET3 binds mainly to ETBR of endothelium and fulfill the third essential sequential step of the KIT-ET3-NO pathway. Upon binding of ET3 to ETBR on endothelium, cytosolic Ca^2+^ increases [11,12], leading to cell-specific Ca^2+^-dependent eNOS activation [10–12], and subsequent temporally- and spatially-precise NO generation, thus fulfill the fourth and fifth steps of the KIT-ET3-NO pathway respectively. The capacity of ET3-ETBR signaling in eNOS activation and NO generation is well documented *in vivo* [39,40] and *in vitro* [12,38,41–43]. The physiological role of ET3-ETBR signaling-mediated NO in endothelium function is well established and recapitulated below. ET3 has been shown to stimulate cGMP via ETBR-mediated NO generation in isolated rat glomerulus and cultured rat mesangial cells [43] and in bovine endothelial cells [41,12]. The NO generated by the KIT-ET3-NO pathway in endothelium can diffuse into VSMCs, initiate the NO/cGMP pathway, and activate soluble guanylate cyclase to generate cGMP, thereby activate protein kinase G leading to vasodilation and many other endothelium functions for maintaining vascular homeostasis, thus fulfill the sixth and the last step of the KIT-ET3-NO pathway. The KIT-ET3-NO pathway-mediated NO generation in endothelium in turn negatively regulates acetylcholine release [76], ET1 signaling [20,21], and ET1 synthesis [22] to ensure a tightly regulated loop of the KIT-ET3-NO pathway. Simply put, ET3 plays a unique role of mediating cross talk between SCF-KIT signaling and NO/cGMP pathway in blood vessels. The critical physiological role of the KIT-ET3-NO pathway in increasing endothelium-dependent NO generation for compensating normal physiological and pathological conditions are detailed below.

### The KIT-ET3-NO pathway plays critical roles in endothelium function: Endothelium depends on the KIT-ET3-NO pathway to fulfill the high demand (exceeding basal level) of nitric oxide generation in coping with pregnancy, aging, and atherosclerosis

Blood vessels in surgical specimens obtained under anesthesia from normal human patients without cardiovascular diseases represent the physiological condition of minimal demand for endothelium-dependent NO generation beyond basal level, hence not useful for our *in vivo* studies. To assess the physiological role of the KIT-ET3-NO pathway in maintaining vascular homeostasis, we first examine the physiological roles of the individual four key molecules (i.e. ETBR, ET3, SCF, and KIT). The critical physiological roles of ETBR and ET3 as individual molecules in endothelium-dependent NO generation for maintaining vascular homeostasis when the demand for NO is higher than basal level during pregnancy [25–27] and for compensating physiological aging [33] respectively have been reported and are presented earlier (refer to Introduction).

To investigate the role of SCF-KIT signaling in endothelium-dependent NO generation for maintaining vascular homeostasis, we can either establish a KIT-knockout animal model or administer highly specific KIT inhibitor and examine the phenotypes or clinical outcomes respectively, the former is difficult because homozygous loss-of-function *KIT* mutation results in death before birth due to severe anemia [77], and the latter has taken place already, namely the Philadelphia chromosome-positive chronic myelogenous leukemia (CML) clinical trials comparing nilotinib (Tasigna^®^, Novartis Pharmaceuticals) [78–80] with imatinib (Gleevec^®^, Novartis Pharmaceuticals) [64,81,82]. Rather than reinventing the wheel, we will present the nilotinib CML clinical trials and analyze all relevant published data for evaluation of the unexpected, still unexplained, and serious vascular side effects, which can potentially offer critically important insight.

Nilotinib is a second generation, and imatinib a first generation, of highly selective targeted drugs against the same four targets of BCR-ABL (and ABL), PDGFR-α, PDGFR-β, and KIT. The nilotinib CML clinical trial design has no placebo arm, instead it has a second arm using imatinib. The high frequency and severity of adverse vascular event (AVEs) in patients treated with nilotinib, but not imatinib, are unexpected and currently unexplained indicating gaps in current knowledge thus warrant evaluation. To start, we take a close look at the nature of AVEs and re-analyze all available data. The prescribing information of nilotinib reports that with a median time on nilotinib therapy for 60 months in clinical trials, the overall incidence of AVEs occurred in 9.3% and 15.2% of all patients enrolled to the nilotinib 300 and 400 mg twice daily arms respectively, and in 3.2% of all patients enrolled to the imatinib arm [83]. The AVEs of nilotinib has been independently reported from single and multi-institutional retrospective studies [84–91], as a prospective study [92], as correspondence, letters, or comments (93–95), and again in recent review articles [96–99]. The AVEs from above reports are nontrivial, some are life-threatening and many require surgery. They include two cases of sudden death [84,93], peripheral arterial occlusive disease (PAOD) [84,85,87,89,90,92–94], myocardium infarction [84,87,90], stroke [85,88,90], carotid artery stenosis [90], bilateral renal artery stenosis [86], coronary artery disease [85,90], spinal infarction [84], subdural hematoma [84], Raynaud syndrome [85], pulmonary emboli [85], and iliac artery stenosis [90]. In a single institution study, the projected 10-year actuarial probability of remaining PAOD-free is 100% in imatinib group and only 67% in the nilotinib group [87]. A prospective study again demonstrates high incidence of AVEs despite a short 30-months duration of nilotinib therapy, and the relative risk for PAOD is calculated to be 10.3 in patients receiving first-line nilotinib as compared with patients receiving first-line imatinib [92]. The above ample reports from multiple independent investigators and institutions indicate valid observations and widespread concerns regarding nilotinib-induced AVEs. The dose-dependent nature, high frequency, and high relative risk for PAOD speak against coincidence. The serious life-threatening nature is alarming. The causal relationship between nilotinib and the AVEs is beyond doubt, but the mechanism so far remains unknown.

The first step towards investigation of nilotinib side effects is to identify the target tissue that incurred drug-induced insult by nilotinib, but not by imatinib. The clinical presentation and objective medical evaluation indicate that nilotinib-induced AVEs are proatherogenic in nature [88,94–99] leading to acceleration of pre-existing atherosclerosis and development of new atherosclerosis in vulnerable patients with underlying risk factors with or without prior AVEs before nilotinib treatment.

The major adverse events are “vascular” and “proatherogenic” in nature [88,94–99], so, the most possible “relevant wild-type target tissue” that suffers nilotinib-induced injury leading to its impairment would be endothelium as endothelium-dependent NO generation confers critically important anti-atherogenic protection (refer to Introduction for background).

The second step is to identify the “relevant wild-type target molecule” on endothelium, which is inhibited specifically by nilotinib, but not by imatinib. The high frequency of severe AVEs in the nilotinib arm but not in the imatinib arm in CML trials may appear puzzling on the surface, but upon close examination, it provides a valuable clue. Clinical trial results of high AVEs by nilotinib in one arm and low AVEs by imatinib in the other arm strongly indicate that the IC_50_ of the relevant yet-unknown “wild-type target molecule” by these two drugs must be vastly different.

Since ABL and BCR-ABL has never been shown to be expressed in endothelium, inhibition of ABL (or BCR-ABL) is unlikely to account for the nilotinib-induced AVEs. The other three main targets of nilotinib and imatinib, namely KIT, PDGFR-α, and PDGFR-β, are expressed on endothelium, so comparison of IC_50_ of these three main targets in wild-type form, not mutated form, by nilotinib and imatinib represents the necessary and sufficient step towards ruling out irrelevant targets and narrowing down to the single critical “relevant wild-type target molecule”, the function of which is essential in preventing nilotinib-induced AVEs.

There are significant structural differences between nilotinib and imatinib with a Daylight-fingerprint-Tanimoto similarity coefficient of 0.6 [79]. Both nilotinib and imatinib are ATP-competitive inhibitors and bound within the ATP-binding pocket of KIT, PDGFR-α, and PDGFR-β. Imatinib and KIT interaction has been well analyzed [81]. Nilotinib also binds to a catalytically inactive conformation (DFG-out) of the activation loop, and prevents KIT from becoming activated [78,80]. We first examine nilotinib’s capacity in inhibition of proliferation (IC_50_-proliferation) and inhibition of tyrosine-autophosphorylation (IC_50_-autophosphorylation) of the three main targets (i.e. KIT, PDGFR-α, and PDGFR-β) in their “wild-type” form without mutation in the presence of respective ligand. The common approach is to establish Ba/F3 cells that are transfected with specific targets for IC_50_ testing, and these cells are conventionally designated as “target-(Ba/F3)” cells.

It is critically important to point out that IC_50_ of a given drug against a specific mutated target can be drastically different from the IC_50_ against the wild-type counterpart. A single missense mutation in targets can potentially result in drastic changes in 3-dimensional conformation and binding affinity thus lead to drastically different IC_50_ and tumor response of a given drug. For example, Weisberg *et al*. [78] reported that IC_50_-proliferation of p210-BCR-ABL-(Ba/F3) by nilotinib is 25 nM, while the IC_50_-proliferation of T315I-BCR-ABL-(Ba/F3) by nilotinib is >10,000 nM due to a mutation resulted in one single amino acid substitution. So, the IC_50_ of “mutated target” bears no relevance to adverse side effects inflicted on normal tissues.

The prescribing information of nilotinib [83] reports “IC_50_ of c-kit” to be “210 nM”, but fail to specify the target (i.e. wild-type human KIT *vs* mutated human KIT), hence this IC_50_ value of “210” is meaningless. Further review of published data reveals that IC_50_-proliferation of the GIST882 cell line, which harbors K642E-KIT mutation in exon 13, to be 200±13 [78], very close to the value reported in the prescribing information of nilotinib [83]. The IC_50_ of mutated KIT by nilotinib using the GIST882 cell line most definitely does not represent the IC_50_ of wild-type human KIT, hence is not helpful in the investigation of nilotinib side effects. In their effort to characterize pediatric GISTs, Agaram *et al*. [100] transfected wild-type human *KIT* into Ba/F3 cells, and reported IC_50_-proliferation of wild-type-KIT-(Ba/F3) by nilotinib and imatinib to be 35 nM and 3,132 nM respectively, and these vastly different IC_50_ of KIT by nilotinib and imatinib represent the key information to the understanding of the nilotinib-induced AVEs as delineated below.

Nilotinib is a potent inhibitor of BCR-ABL (IC_50_ = 25 nM) as intended, but what is not well publicized or even recognized is that nilotinib is also an extremely potent specific inhibitor of wild-type KIT (IC_50_ = 35 nM) exhibiting an astounding 90-fold lower IC_50_ than that of imatinib (IC_50_ = 3,132 nM) [100]. In keeping with the proliferation assay, inhibition of wild-type KIT tyrosine phosphorylation by immunoprecipitation and Western blot assays showed equally dramatic results demonstrating that nilotinib, at 100 nM, the wild-type KIT tyrosine phosphorylation was inhibited by more than 50%, while imatinib at 5,000 nM, the wild-type KIT tyrosine phosphorylation persisted although much reduced [100].

This high IC_50_-proliferation as well as high IC_50_-autophosphorylation of imatinib against wild-type-KIT-(Ba/F3) is well reflected clinically as well. Pediatric GISTs typically lack mutations in *KIT* or *PDGFR-A*, often over-express wild-type *KIT*, and these GISTs exhibiting over-expressed wild-type KIT are resistant to imatinib [100]. The very high IC_50_-proliferation of wild-type-KIT-(Ba/F3) of 3,132 nM by imatinib and the *in vivo* clinical experience of resistance of wild-type GISTs to imatinib indicate a low probability of inhibition of wild-type KIT on all human normal tissues including endothelium by imatinib, hence a low probability of causing AVEs in the imatinib arm. The contrasting extremely low IC_50_-proliferation of wild-type-KIT-(Ba/F3) of 35 nM by nilotinib strongly indicates a very high probability of inhibition of wild-type KIT on all human normal tissues including endothelium by nilotinib, hence a very high probability of causing AVEs in nilotinib arm. The extremely low IC_50_-proliferation of wild-type-KIT-(Ba/F3) of 35 nM by nilotinib in comparison with the very high IC_50_-proliferation of wild-type-KIT-(Ba/F3) of 3,132 nM by imatinib, an astonishing 90-fold difference, explains fully the clinical outcome of significant AVEs frequency difference of 15.2% *vs*. 3.2% in nilotinib arm *vs*. imatinib arm respectively as reported in the nilotinib CML trials [83].

The next step is to determine whether the other two wild-type targets play any role in the nilotinib-induced AVEs. (i) Wild-type PDGFR-β. The IC_50_-proliferation of wild-type-PDGFR-β-(Ba/F3) by nilotinib and imatinib were reported to be 57 nM and 39 nM respectively [78]. (ii) Wild-type PDGFR-α plus wild-type PDGFR-β. The IC_50_-autophosphorylation of (wild-type-PDGFR-α plus wild-type-PDGFR-β)-(A31) by nilotinib and imatinib were reported to be 71 nM and 74 nM respectively [78]. With comparable IC_50_ by nilotinib and imatinib as shown above, inhibition of wild-type PDGFR-α and or wild-type PDGFR-β is highly unlikely to account for the significant AVEs frequency difference of 15.2% *vs*. 3.2% in nilotinib arm *vs*. imatinib arm respectively [83].

In summary, above evaluation of nilotinib CML trials and re-analysis of IC_50_ of wild-type KIT, PDGFR-α, and PDGFR-β as described above reveal two important facts regarding the AVEs observed in nilotinib CML clinical trials. (i) Being an extremely potent highly selective inhibitor against wild-type KIT (IC_50_ = 35 nM), nilotinib can dose-dependently and highly selectively abolish KIT-mediated signaling in endothelium and results in endothelium impairment. (ii) Wild-type KIT, but not wild-type PDGFR-α and or wild-type PDGFR-β, represents the “relevant wild-type target molecule” in endothelium that is specifically inhibited by nilotinib (IC_50_ = 35 nM), but not by imatinib (IC_50_ = 3,132 nM). This inhibition of a yet-unknown signaling/pathway mediated by KIT in endothelium accounts for the nilotinib-induced AVEs in vulnerable patients enrolled in nilotinib arm [83–99]. In another word, nilotinib-induced AVEs predict a yet-unknown KIT-mediated signaling/pathway in endothelium, which is essential in preventing nilotinib-induced AVEs.

The vulnerable CML patients with and without prior AVEs present with acceleration of pre-existing atherosclerosis and rapid onset of developing new atherosclerosis respectively, followed by serious AVEs after a short nilotinib treatment for only 30 to 60 months [83–99]. This unusual clinical presentation plus objective medical evaluations indicate that nilotinib-induced AVEs are proatherogenic in nature [88,94–99] due to loss of anti-atherogenic protection.

Intact healthy endothelium confers anti-atherogenic protection mainly by generating adequate endothelium-dependent NO, which plays multiple critical roles including inhibiting platelet and leukocyte aggregation and maintaining the VSMCs in a non-proliferative state, thus prevent acceleration of pre-existing or developing new atherosclerosis [2,13,16,18] (refer to Introduction). The vulnerable CML patients who suffered nilotinib-induced AVEs most likely depend on a yet-unknown SCF-KIT signaling-mediated pathway in endothelium as their last reserve to meet their high demand of endothelium-dependent-NO generation to maintain sufficient NO bioavailability and vascular homeostasis. In another word, a yet-unknown SCF-KIT signaling-mediated pathway in endothelium helps these vulnerable patients compensating for their underlying pathophysiology thus preventing acceleration and development of atherosclerosis. Inhibition of KIT by nilotinib results in endothelium impairment, undermines these patients’ anti-atherogenic protection, and deprives these vulnerable CML patients of their last reserve of endothelium-dependent-NO generation.

The KIT-ET3-NO pathway qualifies to be the yet-unknown KIT-mediated endothelium-dependent NO generation signaling/pathway predicted by the nilotinib-induced AVEs. In retrospect, with hindsight, the sequence of events are as follows. Nilotinib, being a potent highly selective inhibitor of wild-type KIT (IC_50_ = 35 nM), inhibits the first step of the KIT-ET3-NO pathway and results in endothelium impairment. Inhibition of SCF-KIT signaling abolishes induction of ET3. Deprivation of ligand ET3 abolishes ETBR activation in endothelium, thus compromises the capacity to fulfill the high demand of endothelium-dependent NO and leads to insufficient NO bioavailability. Insufficient NO bioavailability in term leads to loss of anti-atherogenic protection, renders intimal and medial thickening and vascular stiffness in arterial walls, followed by acceleration of pre-existing atherosclerosis and development of new atherosclerosis, thus induces the clinical manifestation of rapid development of serious AVEs in vulnerable CML patients.

In summary, in addition to inhibiting BCR-ABL (IC50 = 25 nM), nilotinib is also a potent and specific inhibitor of wild-type KIT (IC_50_ = 35 nM) as illustrated above. The nilotinib-induced AVEs in CML clinical trials provide solid evidences indicating a critical role of KIT in fulfilling the high demand of endothelium-dependent NO generation when the demand exceeds basal level for anti-atherogenic protection in subjects harboring underlying pathophysiology (e.g. atherosclerosis).

Taken together, pregnancy, aging, and atherosclerosis share one common character of high demand for endothelium-dependent NO generation, and it is utterly unlikely for endothelium to evolve three different mechanisms for the sole purpose of positive modulation of endothelium-dependent NO generation. So, what we have learned about the critical roles of ETBR, ET3, and SCF-KIT signaling in fulfilling the high demand of endothelium-dependent NO generation in pregnancy, aging, and atherosclerosis respectively are complementary, and these isolated evidences converge to support the physiological role of one unified pathway, the KIT-ET3-NO pathway.

We cited many references for background and completeness. Many of these reports were considered seemingly unrelated, isolated, and standing-alone facts, many were not included in review articles, thus became forgotten over the years. Our studies demonstrate that the SCF-KIT signaling can induce synthesis and secretion of ET3, and these results enable us to connect isolated supporting data from literature and lead to the discovery of the KIT-ET3-NO pathway. Excluding all background references, the following four studies constitute the supporting essential data from literature to help demonstrating the critical physiological role of the KIT-ET3-NO pathway in endothelium function: (**i**) nilotinib CML clinical trials and unexpected adverse vascular events. Although we cited all related publication [83–99], they should be considered as a single source of supporting essential data, (**ii**) molecular characterization of pediatric GISTs by Agaram *et al*. 2008 [100], (**iii**) studies on pregnant rats by Mazzuca *et al*. 2013, [26], and (**iv**) studies on senescent rats by Lattmann *et al*. 2005 [33].

In conclusion, integration of background information, above rationales, supporting essential data from the above four studies in literature, and our results together demonstrate that human endothelium depends on the KIT-ET3-NO pathway in fulfilling the high demand of the endothelium-dependent-NO generation (exceeding basal level) in coping with pregnancy, aging, and atherosclerosis for maintaining vascular homeostasis.

#### Comparison between acetylcholine and the KIT-ET3-NO pathway in endothelial nitric oxide synthase (eNOS) activation

Intact functional endothelium is obligatory for the acetylcholine-induced relaxation of VSMCs [19]. Acetylcholine-induced vasodilation involves binding of acetylcholine to muscarinic receptor on VSMCs to induce Ca^2+^ release from VSMCs intracellular storage site. The Ca^2+^ thus induced can efflux from VSMCs into the intercellular space followed by influx of Ca^2+^ into the endothelium leading to eNOS activation. The KIT-ET3-NO pathway involves cellular communication and activation of SCF-KIT signaling and induction of ET3, which binds to ETBR leading to the release of Ca^2+^ directly within endothelium for eNOS activation without involvement of Ca^2+^ storage in VSMCs, Ca^2+^ efflux from VSMCs into intercellular space, or Ca^2+^ influx into the endothelium. The KIT-ET3-NO pathway, which operates under a distinctly different mechanism, is non-redundant and cooperative with acetylcholine in fulfilling the high demand of endothelium-dependent NO generation for maintaining vascular homeostasis.

### Human skin and colon myenteric plexus exhibit activation of the first two steps of the KIT-ET3-NO pathway *in vivo* upon the physiological stimuli of chronic heavy sun-exposure and prolonged fasting respectively

Many diverse cell types express KIT (refer to Introduction for a complete list). The next logic question is: In addition to endothelium, does the KIT-ET3-NO pathway also plays critical physiological roles in other KIT- and SCF-expressing cell functions? We studied the first two of the six essential consecutive steps of the KIT-ET3-NO pathway, namely the activation of SCF-KIT signaling and induction of ET3 *in vivo* in two human systems upon two unique physiological stimuli respectively. The rationale for these studies is three-fold: (1) To further strengthen our finding of induction of ET3 by SCF-KIT signaling *in vivo*. (2) After establishing the critical physiological role of the KIT-ET3-NO pathway in endothelium function, we move on to investigate whether the KIT-ET3-NO pathway represents a unified mechanism underlying other SCF- and KIT-expressing cell functions. (3) These studies set the stage to facilitate further physiological studies in all SCF-expressing and KIT-expressing cell functions in the future.

#### KIT activation and nuclear localization of phosphotyrosine 568/570KIT

The first step is to establish a sensitive and reliable method to detect whether KIT is activated or not *in vivo*. Ligand binding of SCF to KIT results in dimerization of KIT, activation of its intrinsic tyrosine kinase activity, and autophosphorylation of specific tyrosine residues of KIT [66–68]. Taking advantage of this first step of KIT activation, we used six antibodies against the phosphorylated tyrosine peptides of KIT that recognize phosphotyrosine (pY)568/570, pY703, pY721, pY730, pY823, and pY936 respectively for IHC on frozen sections of GISTs exhibiting various degrees of aggressiveness to serve as positive controls, and normal adult human testis placed on the same slide to serve as external negative control. We also used the pY568/pY570 antibody for *in situ* IHC on KIT-expressing melanoma WM793 cells at various time points after SCF stimulation to study the kinetics.

**Fig 2:**
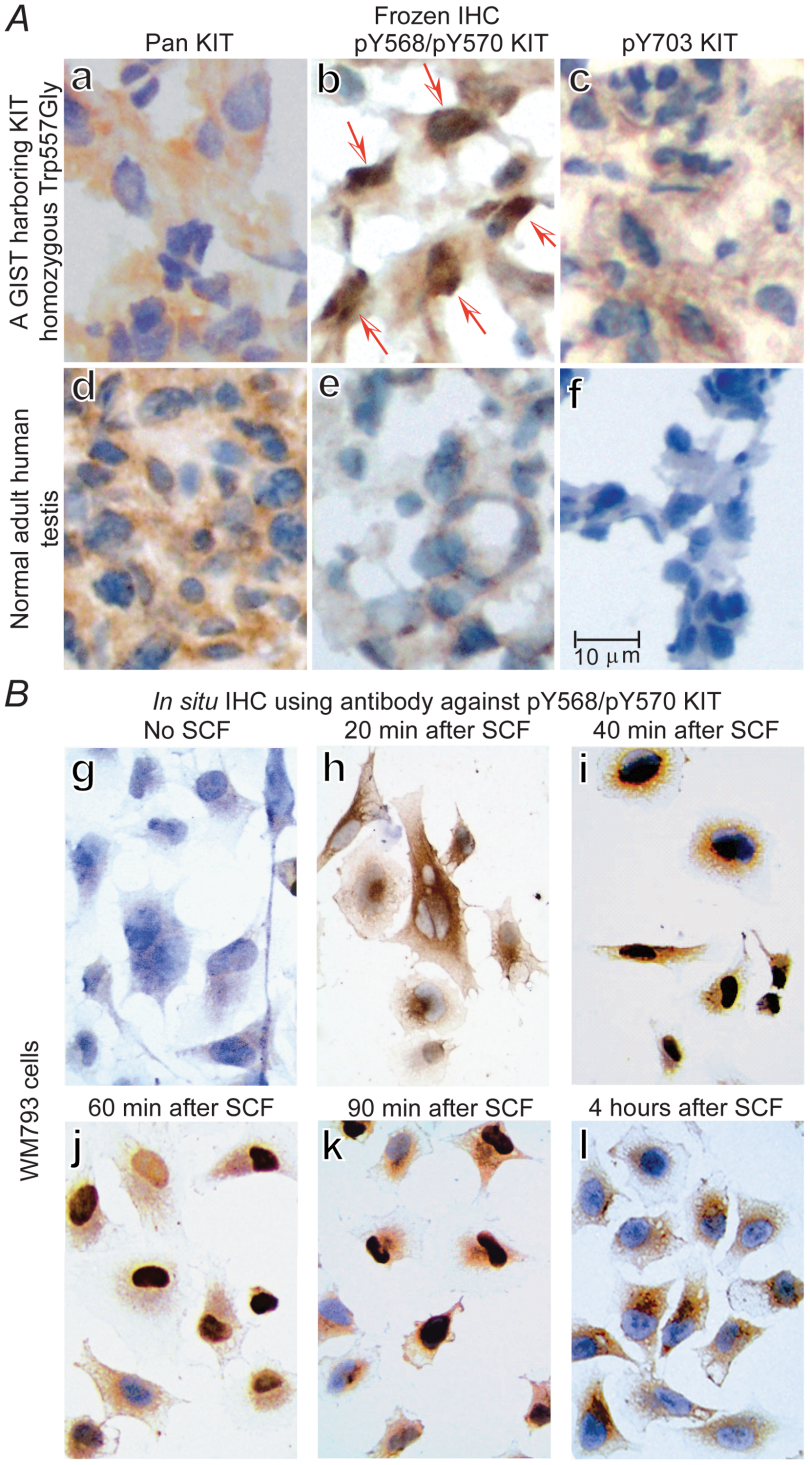
Autophosphorylation, internalization, and nuclear localization of activated KIT with tyrosine phosphorylation at 568/570 (pY568/pY570KIT). (***A***), IHC of frozen sections of an aggressive GIST (*a-c*) and a normal human adult testis as external control (*d-f*) using pan-KIT antibody (*a* and *d*), pY568/pY570KIT antibody (b and *e*, red arrow indicates nuclear localization), and pY703KIT antibody (*c* and *f*) respectively. (***B***), *In situ* IHC to assess kinetics of SCF-induced nuclear translocation of pY568/pY570KIT using WM793 melanoma cells cultured in 4-well chamber tissue culture treated glass slides. Control (*g)* without SCF stimulation, after addition of SCF to culture media, the nuclear localization of pY568/pY570KIT increases progressively (*h-j*) in more than 90% of WM793 cells, reaches a plateau about 40–60 minutes (*i* and *j*), begins to decrease at 90 minutes (*k*), and is completely absent in nucleus with relocation back to the cytoplasm at 4 hours, some residual cytoplasmic staining remains visible (*l*).

Three-dimensional structure analysis indicates that Y586KIT is the primary autophosphorylation site upon SCF binding [81]. The pY568/pY570KIT antibody recognizes both pY568KIT and pY570KIT because of their close proximity, so positive staining can represent pY568 alone or pY570 alone or both pY568 and pY570. The polyclonal pan-KIT antibody recognizing the *C*-terminal end of non-phosphorylated KIT shows positive cytoplasmic staining and negative nuclear staining in GISTs (Fig 2Aa) and normal adult human germ cells (Fig 2Ad). In sharp contrast, the antibody against pY568/pY570KIT shows distinctive strong positive nuclear staining in all GISTs, and interestingly, the percentage of positive nuclear staining is proportional to the aggressiveness of the GIST (data not shown). One typical example of an aggressive GIST exhibits 57% nuclear localization of pY568/pY570KIT (Fig 2Ab, red arrows). No nuclear staining can be identified on lymphocytes (internal control) or adult testis (external control) (Fig 2Ae). Antibody against pY703KIT on GIST (Fig 2Ac) or testis (Fig 2Af) shows no nuclear staining, and the same is true for pY721, pY730, pY823, Y936 antibodies (data not shown).

*In situ* IHC using antibody against pY568/pY570KIT on WM793 cells shows that the control without SCF demonstrates total negative staining (Fig 2Bg). After stimulation with SCF, the activated pY568/pY570KIT begins nuclear localization within 20 minutes (Fig 2Bh), reaches a peak at 40–60 minutes in more than 90% of WM793 cells (Fig 2Bi and 2Bj), begins to decrease after 90 minutes (Fig 2Bk), and is completely absent from nucleus with relocation back to cytoplasm at 4 hour-time point with some residual cytoplasmic staining remains visible (Fig 2Bl).

Above results demonstrate that KIT activation is tightly regulated by ligand availability, transient, and local. Above results also demonstrate that IHC using antibody recognizing pY568/pY570KIT represents a sensitive and reliable method of detecting the *in vivo* activated KIT. Nuclear localization of cytoplasmic membrane receptors leading to induction of downstream target genes is infrequent but has been reported [101,102]. Nuclear localization of pY568/pY570KIT is unexpected [66–68,103,104], suggests potential transcriptional capacity, and warrants investigation, but its biological significance is beyond the scope of this present study. IHC staining of pY568/pY570KIT and nuclear localization of pY568/pY570KIT are utilized in this study as indicators of “*in vivo* activated KIT” status, and are applied to the rest of experiments described below.

#### Activation of SCF-KIT signaling and concomitant parallel induction of endothelin-3 (ET3) in proportion to the extent of sun-exposure in human skin

**Fig 3:**
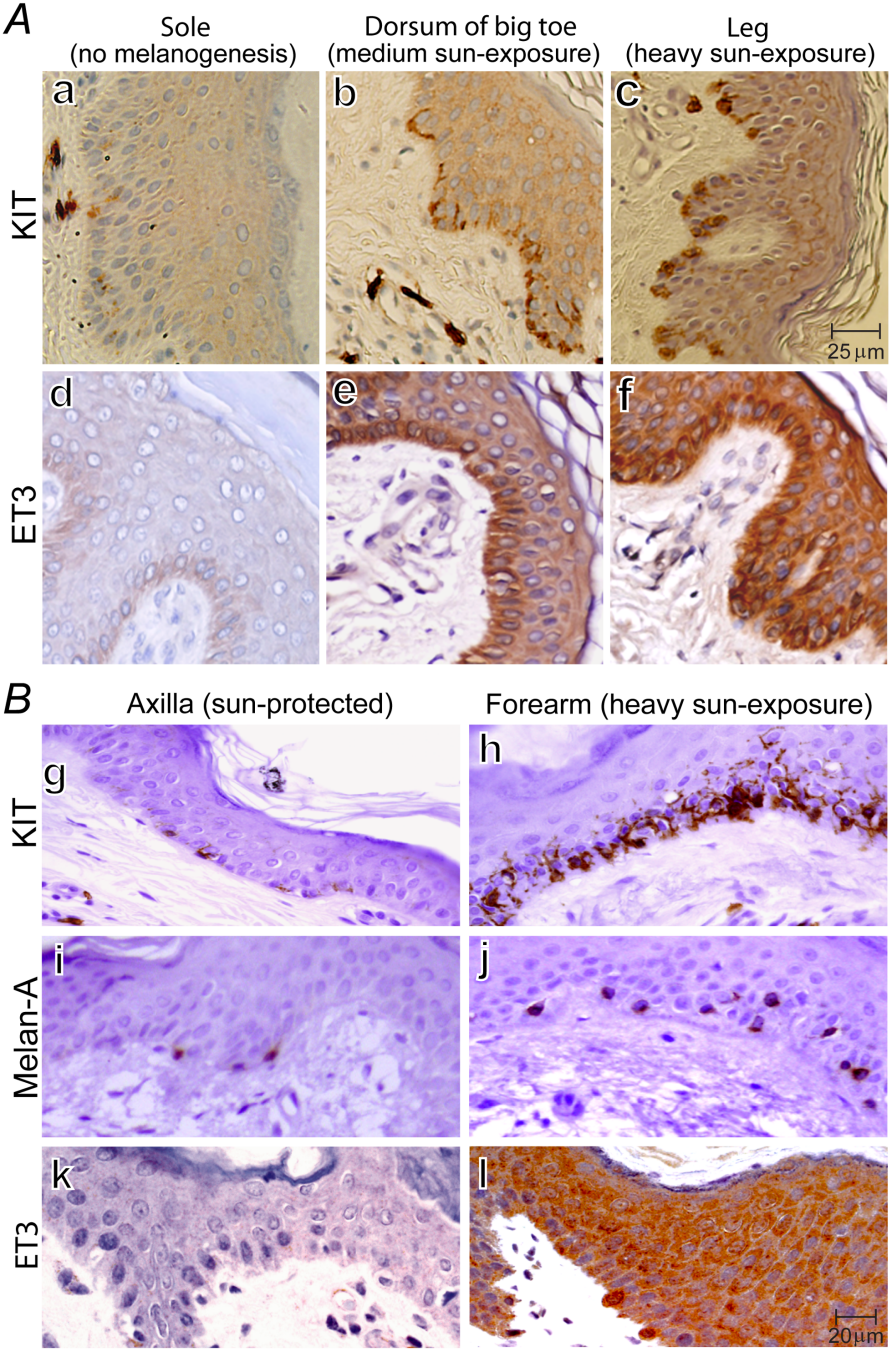
KIT activation & up-regulation, concomitant parallel induction of ET3, KIT^+^Melan-A^–^- progenitor cells, and melanocyte regeneration in proportion to sun-exposure. (***A***), IHC of KIT and ET3 on serial sections of human skin specimen obtained from a lower extremity-amputation. Sole represents active suppression of melanogenesis (*a* and *d*), dorsum of big toe represents intermediate sun-exposure (*b* and *e*), and lateral lower leg represents heavy sun-exposure (*c* and *f*). (***B***), IHC of KIT, Melan-A, and ET3 on serial sections of human skin punch biopsy specimens obtained from sun-protected axilla (*g*, *i*, *k*) and chronic heavy sun-exposed forearm (*h*, *j*, *l*) from the same individual. Lymphocytes serve as internal negative control for KIT, ET3 and Melan-A; mast cells serve as internal positive control for KIT. Together, these images demonstrate that human skin exhibits sun-exposure-dependent up-regulation of KIT (*a-c*) and concomitant parallel sun-exposure-induced increasing induction of ET3 (*d-f*). Chronic sun-exposure induces intense dendritic pattern of KIT expression as well as a large increase in the number of KIT-expressing-cells in the basal layer (*h*) consisting of KIT^+^Melan-A^+^ mature melanocytes (*j*) and KIT^+^Melan-A^–^melanocyte progenitor cells as evidenced by the difference between (*h*) and (*j*).

The SCF-expressing keratinocytes serve as first line defense against environmental stimuli of sun-exposure and receive in-puts from multiple systems (e.g. nervous and endocrine). Human skin specimens are readily available from unused to-be-discarded surgical specimens. We studied human skin specimens with varying extent of sun-exposure and compared them within the same individuals (Fig 3). Typical examples of skin specimens from the sole, dorsum of big toe (moderate sun-exposure), and lateral lower leg (chronic heavy sun-exposure) from the same individual (a leg-amputee) are shown in Fig 3A. Negative staining of KIT and ET3 on lymphocytes serve as internal negative control, and the positive KIT staining on mast cells serve as internal positive control. Sole manifests minimal expression of KIT and total absence of ET3 (Fig 3Aa and 3Ad), and they are used as baseline control for comparison. We observed progressive increase of KIT by IHC (Fig 3Aa–3Ac), progressive activation of KIT by pY568/pY570KIT IHC (data not shown), and concomitant parallel increasing induction of ET3 *in vivo* (Fig 3Ad–3Af) in proportion to the extent of sun-exposure. Sole has minimal sun-exposure, moreover, melanogenesis is actively suppressed in the sole [105,106], so, our finding of minimal KIT expression and total absence of ET3 (Fig 3Aa and 3Ad), serves as an additional independent support of the critical role of SCF-KIT signaling and ET3 in melanogenesis.

We next obtained punch biopsy skin specimens from chronic heavy sun-exposed skin (dorsum of forearm) and sun-protected skin (axilla) from five volunteers and compared KIT, Melan-A and ET3 expression of matched pairs within the same individual. A typical such example is shown in Fig 3B. Less than 11% of the cells in the basal layer of sun-protected skin (axilla) show positive staining of KIT (Fig 3Bg), whereas more than 85% of the cells in the basal layer of chronic heavy sun-exposed skin (dorsum of the forearm) exhibit dendritic pattern of intense KIT staining indicating up-regulation of KIT expression (Fig 3Bh) and activation of KIT by positive pY568/pY570KIT IHC (data not shown). The KIT^+^ cells were 7.2± 0.12-fold more abundant in the chronic heavy sun-exposed skin than that in the sun-protected skin (*P* < 0.0001) (Fig 3Bh vs. 3Bg). ET3 is completely absent in sun-protected axilla (Fig 3Bk), while robust induction of ET3 is obvious as demonstrated by the intense ET3 staining (Fig 3Bl). ET3 can be visualized surrounding both melanocytes and keratinocytes (*f* and *l*). Mature melanocytes are conventionally defined as “KIT-expressing-Melan-A-producing” cell in the basal layer of skin. Comparison of Melan-A^+^ cells in biopsy pairs demonstrated a three-fold increase by chronic heavy sun-exposure (Fig 3Bj vs. 3Bi). There exists a big discrepancy in the basal layer of chronic heavy sun-exposed skin (forearm) between KIT^+^ (Fig 3Bh) and Melan-A^+^ (Fig 3Bj) cells indicating emergence of abundant KIT^+^ Melan-A^–^cells accounting for approximately half of all KIT^+^ cells. The emergence of abundant KIT^+^ Melan-A^–^progenitor cells and a three-fold increase of KIT^+^ Melan-A^+^ mature melanocytes indicate robust and on-going melanocyte regeneration.

In conclusion, above *in vivo* studies (Fig 3) demonstrate that chronic heavy sun-exposure results in activation of SCF-KIT signaling with progressive parallel increasing induction of local ET3 synthesis and secretion (the first two steps of the KIT-ET3-NO pathway) in proportion to the extent of sun-exposure in the basal layer of human skin.

#### Activation of KIT and concomitant parallel induction of endothelin-3 (ET3) in myenteric plexus of human colon post fasting

**Fig 4:**
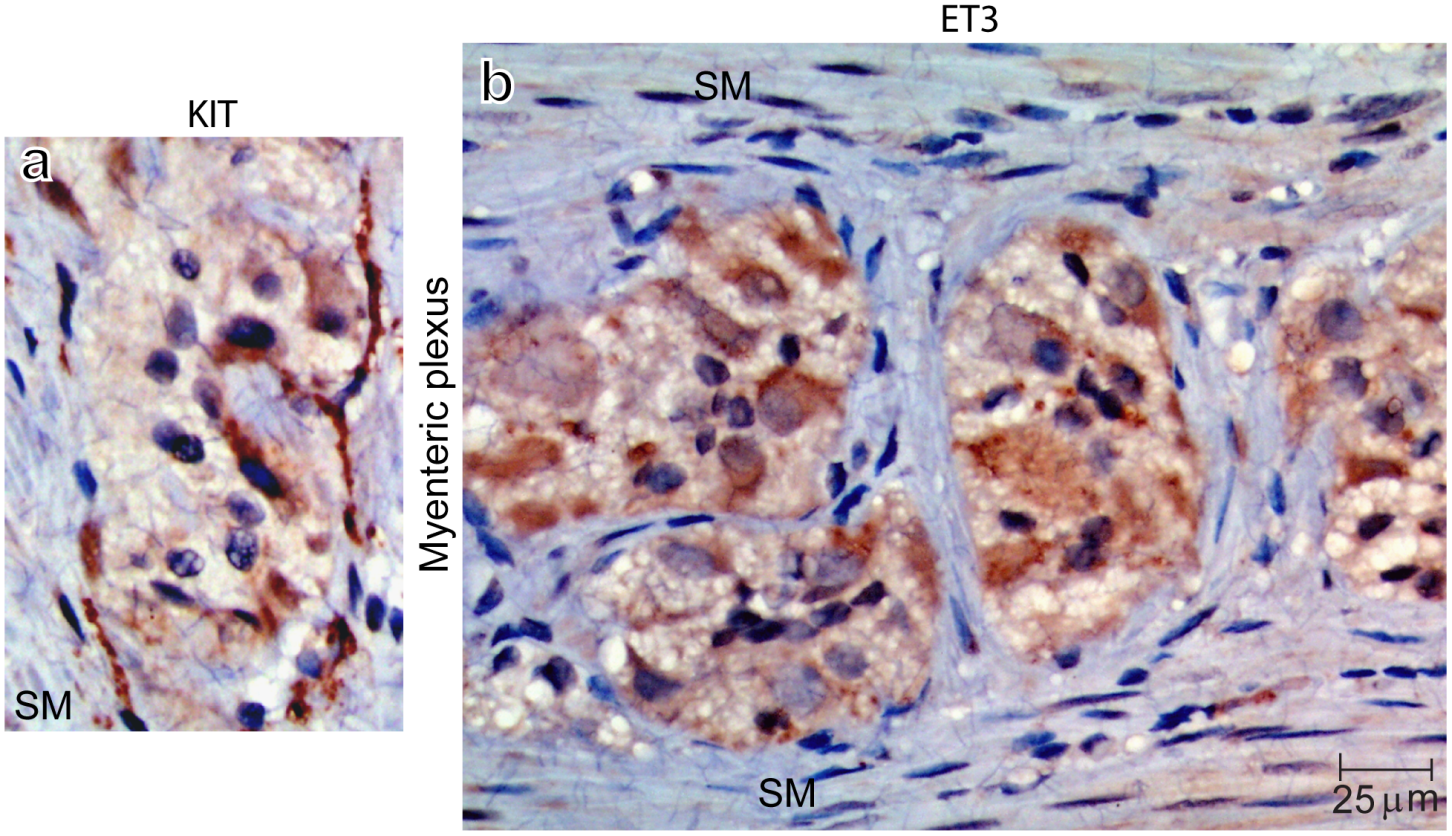
Immunohistochemical studies on human colon myenteric plexus demonstrate that 48 hours fasting results in activation of SCF-KIT signaling and concomitant parallel induction of endothelin-3. Human colon specimens post 48 hours fasting demonstrate intense KIT staining in ICCs within the myenteric plexus (*a*), and intense ET3 staining within the myenteric plexus (*b*). In sharp contrast, the surrounding longitudinal and circular smooth muscle cells (SM) show negative ET3 staining.

The myenteric plexus of the colon consists of ICCs and the enteric nervous system. The ICC network is innervated by the enteric nervous system including the nitrergic neurons, and is linked via many processes throughout the enteric ganglia and surrounding longitudinal and circular smooth muscle cells. Enteric neurons express SCF [70] and ICCs express KIT [60,70]. All surgeries are performed after a minimal of one-day bowel preparation (drink clear liquid only) plus a varying period of fasting overnight and varying waiting period before surgery. We are unable to obtain colon specimen immediately after meal without fasting for comparison as it is against surgical principles to operate without bowel preparation. We examined human colon specimens post varying period of fasting and a typical example post 48 hours fasting is shown in Fig 4. Within the myenteric plexus, intense KIT staining (Fig 4a) identifies ICCs. Positive pY568/pY570KIT IHC staining and nuclear localization confirm that KIT is activated (data not shown). Intense ET3 staining (Fig 4b) indicates induction of ET3 within the myenteric plexus contrasting sharply with the negative ET3 staining in surrounding longitudinal and circular smooth muscle cells (Fig 4b, SM). Enteric neurons can be distinguished from ICCs by their differences in morphology and cell size. The induction of ET3 is progressively increased in proportion to the length of fasting (data not shown).

In conclusion, these results provide *in vivo* evidences indicating KIT activation and concomitant parallel induction of ET3 (the first two steps of the KIT-ET3-NO pathway) in myenteric plexus of human colon after fasting for 48 hours when there is high demand for colonic motility reduction.

#### Human skin and colon myenteric plexus are endowed with the capacity to activate the first five consecutive steps of the KIT-ET3-NO pathway upon chronic heavy sun-exposure and prolonged fasting demanding colonic motility reduction respectively

Upon chronic heavy sun-exposure, communication between SCF-expressing keratinocytes and KIT-expressing melanocytes in human skin can lead to the first and second steps of the KIT-ET3-NO pathway in human skin as demonstrated above (Fig 3). ETBR is expressed in SCF-expressing keratinocytes [107] and melanocytes (refer to Fig 1Ca and 1Ce), such that the SCF-KIT signaling-induced temporally- and spatially- precise secretory ET3 can bind to both keratinocytes and melanocytes thus fulfill the third step of the KIT-ET3-NO pathway. Keratinocytes express nNOS [108], and ET3-ETBR signaling can leads to increase of cytosolic Ca^2+^ [10–12], followed by activation of nNOS in keratinocytes, and lead to temporally- and spatially- precise NO generation, thus fulfill the fourth and fifth steps of the KIT-ET3-NO pathway.

In the event of high demand of ICCs function for negative regulation of colonic motility (e.g. fasting), activation of SCF-KIT signaling in ICCs can lead to the first and second steps of the KIT-ET3-NO pathway in myenteric plexus as demonstrated above (Fig 4). KIT-expressing ICCs express ETBR [109]. ETBR is required for the development of enteric neurons [110,111]. So, the SCF-KIT signaling-induced temporally- and spatially- precise secretory ET3 can bind to the ETBR expressed on both enteric nitrergic neurons and ICCs, thus fulfill the third step of the KIT-ET3-NO pathway. ET3-ETBR signaling can leads to increase of cytosolic Ca^2+^ [10–12], followed by activation of nNOS in nitrergic neurons and lead to temporally- and spatially- precise NO generation, thus fulfill the fourth and fifth steps of the KIT-ET3-NO pathway. The dual requirement of both SCF-KIT signaling and NO in these two systems and three other systems are discussed (refer to the last Section below).

The sixth and last step of the KIT-ET3-NO pathway (i.e. the physiological role) in these two systems is highly likely and of great importance, but a rigorous proof and comprehensive discussion of the physiological role of the KIT-ET3-NO pathway in sun-exposed skin and gastrointestinal motility reduction is beyond the scope of this report and will be reserved for future studies.

#### Implications

Although EPCs is not within the scope of this study, nonetheless it is pertinent to discuss EPCs because of their critical roles in endothelium regeneration and neovascularization for maintaining vascular homeostasis when the demand for endothelium-dependent NO generation exceeds the capacity of existing endothelium and or during post-ischemia repair.

Transplantation of KIT-expressing EPCs demonstrates favorable outcomes of successful grafting, neovascularization [112,113], and functional improvement after ischemic attacks [114]. Ischemia induces matrix metalloproteinase-9 to release soluble SCF from bone marrow stromal cells for binding to KIT on bone marrow EPCs. SCF-KIT signaling is essential for the very first step of EPCs’ activation and exiting from bone marrow quiescent niche [73]. SCF is a potent chemotactic agent for KIT-expressing cells [103]. SCF and KIT are co-expressed in endothelial cells [115] and HUVECs [116] with SCF strategically located on the luminal side. Injury can induce endothelium to produce soluble SCF [117] to serve as homing signaling for EPCs to migrate towards injured/ischemic endothelium. The membrane-bound SCF on the luminal side of endothelium can bind to KIT on EPCs, thus recruit EPCs to injured microvascular endothelium [117].

Post ischemic cardioprotective effects depend on eNOS derived from the donor EPCs [118]. eNOS induction and NO generation are essential for mobilization of stem/progenitor cells [119], NO has been shown to play critical roles in proliferation and differentiation of many progenitor cell types [4,120–124]. Above studies indicate that endothelium regeneration from EPCs requires both SCF-KIT signaling and temporally- and spatially-precise eNOS-derived NO generation from EPCs for the multiple consecutive cellular processes leading to regeneration and revascularization. To seamlessly coordinate independent mechanisms/pathways to connect the above two essential requirements in a timely fashion with precision for the multiple sequential steps during regeneration would be complex if not impossible. On the other hand, a direct connection between these two essential requirements by one pathway appear to make perfect sense, namely activation of SCF-KIT signaling leads directly to timely and precise generation of NO for mediating the multiple sequential steps during the regeneration process. The KIT-ET3-NO pathway represents the most plausible and the only currently known mechanism capable of fulfilling and seamlessly connecting the above two essential requirements. Taken together, we propose that the KIT-ET3-NO pathway most likely plays critical roles in regeneration of endothelium from KIT-expressing EPCs.

The dual requirement of both SCF-KIT signaling and temporally- and spatially-precise NO generation is not limited to endothelium and EPCs. Four additional such examples are briefly described below. (**i**) Erythropoiesis. SCF-KIT signaling and erythropoietin play non-redundant roles in erythropoiesis. Homozygous loss-of-function mutation of *KIT* or *SCF* leads to death before birth due to severe anemia [77]. NO-cGMP signaling is capable of stimulating erythropoiesis *in vitro* and *in vivo* by controlling the expression of multiple lineage-specific transcription factors [125]. (**ii**) Glucose homeostasis. Loss-of-function mutation of *KIT* are associated with diabetes mellitus [126], and SCF-KIT signaling regulates human islet-epithelial cluster proliferation and differentiation [127]. It is truly remarkable that pancreatic islet β-cell-specific over-expression of human *KIT* results in a phonotype of normal glucose homeostasis manifesting improved β–cell function, protection from a diabetes-inducing diet, and rescue of mice harboring mutated *KIT* from developing diabetes [128]. This phenotype of normalization of glucose homeostasis was not observed in β-cell-specific over-expression of human *AKT*-transgenic mice [129]. NO has been shown to exert positive modulation of insulin secretion and anti-apoptosis of β-cells at low concentrations (tens of nanomolar level), and negative modulation of insulin secretion and pro-apoptotic activities at high concentrations (sub-micromolar levels) [5]. These studies show convincing evidences indicating the dual requirement of both SCF-KIT signaling and NO in pancreatic β-cell function in maintaining glucose homeostasis. (**iii**) Negative regulation of gastrointestinal motility. The KIT-expressing ICCs network in the myenteric plexus generates slow waves, functioning as the pacemaker [130–133], and coordinates colonic motility [131,134,135]. SCF-KIT signaling plays a primary role in ICCs function in gut motility regulation [136–138]. The ICCs network is normally under basal (also referred to as spontaneous or tonic) inhibition by NO [134,139–141] manifesting a basal inhibitory relaxation interval between dominant rhythmic colonic migrating motor complexes (CMMCs). The relaxation interval between CMMCs of nNOS-knockout mice is significantly shorter than that of wild-type controls resulting in higher CMMCs frequency and increased colonic motility [135]. These studies provide convincing evidences indicating the dual requirement of both SCF-KIT signaling and NO in negative modulation of gastrointestinal motility. The mechanism of negative regulation of gastrointestinal motility has been a focus of debate because ICCs can coordinate but cannot produce NO while nitrergic enteric neurons can produce NO but cannot coordinate gastrointestinal motility. So, discovery of the KIT-ET3-NO pathway helps resolve this enigma. (**iv**) Melanogenesis and melanocyte regeneration in response to sun-exposure. A genome-wide analysis identified SCF mutations as the cause of familial progressive hyperpigmentation or hypopigmentation depending on the nature of mutation being dominant-activating or loss-of-function respectively [142,143]. Anti-KIT antibody has been reported to attenuate melanocyte regeneration [57] and induce melanocyte apoptosis [144,145]. Transgenic expression of SCF [146] or ET3 [147] results in a similar phenotype of combined hyperpigmentation and melanocytosis. Keratinocytes express iNOS and nNOS [108], and induction of NO in keratinocytes plays an essential role in mediating the sun-exposure-induced melanogenesis and melanocyte regeneration for maintaining homeostasis [108,148–150]. Co-culture of keratinocytes and melanocytes show that melanogenesis depends on the NO generated by keratinocytes, and UV-induced pigmentation is delayed following application of a NOS antagonist to the skin [148]. Taken together, neighboring cells can communicate via SCF-KIT signaling, and NO can modulate cell functions by activating NO/cGMP pathway, controlling the expression of transcription factors, or other mechanisms. In addition to endothelium function as demonstrated in our results, it is highly plausible that the KIT-ET3-NO pathway also plays critical physiological roles in many other KIT- and SCF-expressing cell functions, especially in the aforementioned five examples that exhibit dual requirement of SCF-KIT signaling and NO.

## Abbreviations

AVEs: adverse vascular events
BP: blood pressure
CML: chronic myeloid leukemia
CMMCs: colonic migrating motor complexes
ELISA: enzyme-linked immunosorbent assay
eNOS: endothelial nitric oxide synthase
EPCs: endothelial progenitor cells
ET1: endothelin-1
ET2: endothelin-2
ET3: endothelin-3
ETAR: endothelin-A-receptor
ETBR: endothelin-B-receptor
ETBR^–/–^: ETBR-knockout
ECE-1: endothelin-converting enzyme-1
GIST: gastrointestinal stromal tumor
HUVECs: human umbilical vein endothelial cells
ICCs: interstitial cells of Cajal
IHC: immunohistochemistry
iNOS: inducible nitric oxide synthase
nNOS: neuronal nitric oxide synthase
NO: nitric oxide
NOS: nitric oxide synthase
PAOD: peripheral arterial occlusive disease
PDGFR: platelet-derived growth factor receptor
pY: phosphotyrosine
SCF: stem cell factor
UV: ultraviolet
VSMCs: vascular smooth muscle cells
